# Successful treatment of acanthoma fissuratum with intralesional triamcinolone acetonide

**DOI:** 10.1002/ccr3.2708

**Published:** 2020-02-05

**Authors:** Shivaughn Ramroop

**Affiliations:** ^1^ North Central Regional Health Authority Champs Fleur Trinidad and Tobago

**Keywords:** acanthoma fissuratum, granuloma fissuratum, spectacle frame acanthoma, spectacle frame granuloma

## Abstract

Intralesional triamcinolone acetonide is an effective treatment for acanthoma fissuratum, and recognition of this condition can negate the need for unnecessary surgical intervention.

## Graphical abstract

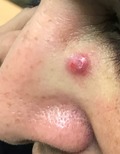

## 1 INTRODUCTION

Acanthoma fissuratum is a relatively rare condition that can mimic skin tumors such as basal cell carcinomas.^1^ We report a case of acanthoma fissuratum that responded well to intralesional triamcinolone acetonide and demonstrate how clinical recognition of this condition can avoid the need for surgical intervention.

## 2 CASE REPORT

**Figure 1:**
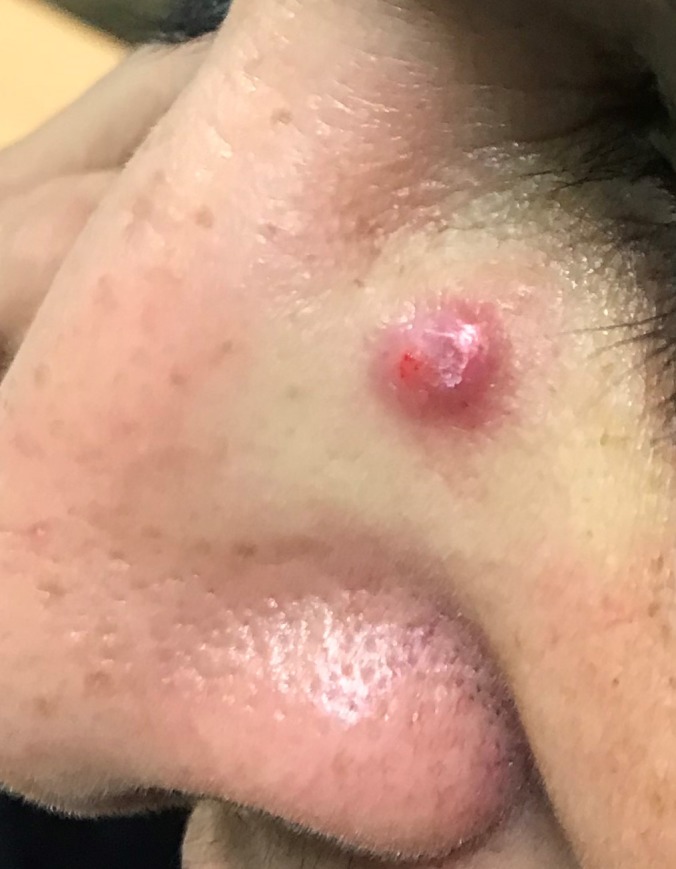
Well‐demarcated nodule with central ulceration

A 52‐year‐old woman presented with a one‐month history of a painful lesion to the upper, left aspect of the nose bridge. It started as a red, flat lesion but progressively became elevated. Examination showed a well‐demarcated, erythematous nodule approximately 1 × 1 cm with superficial, central ulceration (Figure 1). The lesion was very tender on palpation and was located precisely where the patient's glasses made contact with the nose. The patient had her spectacles repaired approximately 6 months prior to development of the lesion, and she described the repaired spectacles as tight fitting. She had no known medical problems and no previous or family history of skin cancer.

A clinical diagnosis of acanthoma fissuratum was made. The patient was treated with an intralesional injection of 0.1 mL triamcinolone acetonide (10 mg/mL) after the lesion was anesthetized with 0.1 mL of 2% lidocaine. She was also advised to have her glasses changed to a lighter, better fitting frame. The main differential was basal cell carcinoma, and excisional biopsy was planned if there was no response to treatment.

**Figure 2:**
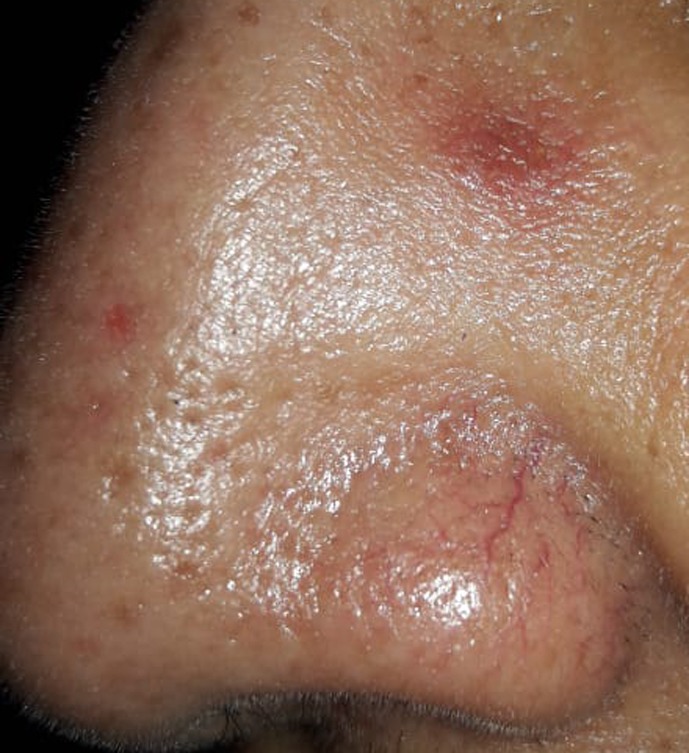
Almost complete resolution 2 wk after intralesional steroid injection

The patient reported significant improvement in pain within 2 days of treatment. The lesion also gradually decreased in size, becoming completely flat with only residual erythema 2 weeks after treatment (Figure 2).

## 3 DISCUSSION

Acanthoma fissuratum is caused by persistent irritation to the skin that results in benign epidermal and dermal hyperplasia.^2^ Despite its synonym, granuloma fissuratum, no granulomas are usually found on histology.^3^ It can present as a papule, nodule, or plaque with central changes.^3^ Though typically associated with poorly fitting eye glasses around the ears or nose,^4^ acanthoma fissuratum has been reported in the oral cavity,^5^ vulva,^6^ and penis.^7^ When found on the nose and ears, the main differential diagnosis is usually basal cell carcinoma.^1^

Typical treatment involves removal of the cutaneous irritant. Intralesional triamcinolone may hasten resolution of the lesion, possibly through its potent anti‐inflammatory effects in the skin. Surgical removal is usually reserved for cases resistant to treatment or when the diagnosis is in doubt.

## 4 CONCLUSION

Intralesional triamcinolone acetonide is an effective treatment for acanthoma fissuratum, and recognition of this condition can negate the need for unnecessary surgical intervention.

## CONFLICT OF INTEREST

The author has no conflicts of interest to declare.

## AUTHOR CONTRIBUTIONS

Shivaughn Ramroop: solely involved in acquiring clinical information, drafting and reviewing the manuscript, and giving final approval of the published version.
